# Germinated brown rice ameliorates obesity in high-fat diet induced obese rats

**DOI:** 10.1186/s12906-016-1116-y

**Published:** 2016-05-23

**Authors:** See Meng Lim, Yong Meng Goh, Norhafizah Mohtarrudin, Su Peng Loh

**Affiliations:** Department of Nutrition and Dietetics, Faculty of Medicine and Health Sciences, Universiti Putra Malaysia, 43400 UPM Serdang, Selangor Malaysia; Department of Veterinary Pre-Clinical Science, Faculty of Veterinary Medicine, Universiti Putra Malaysia, 43400 UPM Serdang, Selangor Malaysia; Department of Pathology, Faculty of Medicine and Health Sciences, Universiti Putra Malaysia, 43400 UPM Serdang, Selangor Malaysia; Research Centre of Excellence, Nutrition and Non-communicable Diseases, Faculty of Medicine and Health Sciences, Universiti Putra Malaysia, 43400 UPM Serdang, Selangor Malaysia; Nutritional Sciences Programme, School of Healthcare Science, Faculty of Health Sciences, Universiti Kebangsaan Malaysia, Jalan Raja Muda Abdul Aziz, 50300 Kuala Lumpur, Malaysia

**Keywords:** Germinated brown rice, Anti-obesity, Diet-induced obese rats, High-fat diet

## Abstract

**Background:**

Germinated brown rice (GBR) is a novel functional food that is high in fiber and bioactive compounds with health-promoting properties. This study aims to evaluate anti-obesity effects of GBR in obese rats fed high-fat diet (HFD).

**Methods:**

Male Sprague-Dawley rats were fed HFD for 8 weeks to induce obesity. The rats were then administrated with GBR where the source of dietary carbohydrate of HFD was replaced by either 25 % GBR, 50 % GBR or 100 % GBR for another 8 weeks. Changes in anthropometry, dietary status, biochemical parameters and histopathology of liver and adipose tissue were measured.

**Results:**

Rats fed with HFD were showed elevation in body weight gain and in white adipose tissue mass compared with rats consumed commercial diet. The GBR administration in 50 % GBR and 100 % GBR were significantly decreased body weight gains and food intakes as well as improved lipid profiles in obese rats. In addition, the administration of GBR  had reduced adiposity by showing declination in white adipose tissue mass, adipocytes size and leptin level concomitantly with a higher ratio of fat excretion into feces. Micro- and macrovesicular steatosis were evidently attenuated in obese rats fed GBR.

**Conclusion:**

These findings demonstrated that GBR exhibited anti-obesity effects through suppression of body weight gain and food intake, improvement of lipid profiles and reduction of leptin level and white adipose tissue mass in obese rats fed HFD.

## Background

Obesity is a condition where abnormal or excessive fat accumulation in the body may affect one person’s health [1]. It is often considered to be a major health threat to disorders such as diabetes mellitus, hypertension, dyslipidemia, coronary heart disease, stroke and several types of cancer as well as increasing the risk of mortality [2]. Recently, about 10.0 % of world adults male and 14.0 % of world adults female were identified as being obese (body mass index, BMI ≥30) in the report of World Health Statistics 2014 [3]. In Malaysia, the prevalence of obese adults was 15.1 % in 2011 [4]. Due to the complex etiology of obesity, therapeutic approaches for long-term obesity management are difficult. Lifestyle intervention includes lowering food intake and increasing physical activity, the main therapeutic strategy for tackling obesity, is usually not so promising, not long-term lasting and require much discipline from the individuals [5]. Anti-obesity drugs, in contrast, are able rapidly to induce weight loss but accompany with adverse effects and user may encounter the problem of regaining weight when discontinued [6].

In recent years, researchers are showing much interest in discovering non-drug strategies such as consuming functional foods or nutraceuticals products as potential therapeutic ways to combat obesity [7]. Numerous studies have examined the anti-obesity effects from various rice species including geminated rough rice [8], germinated brown rice (GBR) [9, 10], black rice [11], wild rice [12] and red mold rice [13]. GBR particularly is quite popular in Asia. It is produced by soaking brown rice in water to induce germination with the germ of about 1 mm long [14]. GBR is not only had intact germ and bran layers that are rich in nutrients, but the hydrolytic enzymes inside the seed have also been activated to produce essential compounds and energy for the formation of seedling during germination [8]. For this reason, it is rich in nutrients such as vitamins, minerals, fibers and bioactive components like γ-aminobutyric acid (GABA), γ-oryzanol and ferulic acid that give numerous protective health effects including antioxidants properties [15, 16], hypocholesterolemic effect [17, 18], hypoglycemic effect [19], anti-colon cancer effect [14], improve Alzheimer’s disease development [20], stimulate osteoblastogenesis [21], and alleviate reproductive system atrophy, dryness and discomfort during menopause [22]. Besides, compared to brown rice, the texture of GBR is found to be less tough and thus better palatability [23].

Previous studies of methanol extract [9] and water extract [10] of GBR demonstrated suppression on body weight gains on mice fed on HFD. Ho et al. [9] reported that methanol extract of GBR was able to reduce adipogenesis in 3T3-L1 adipocytes through the down-regulated expression of adipogenic transcription factors and adipogenic genes. Although few studies have examined the different medicinal properties of GBR and its protective effects in obesity, the information about the use of GBR as a whole instead of extract as a nutraceutical in obesity treatment remains limited. Thus, this study was embarked to investigate the therapeutic effects of GBR as a whole food on diet-induced obese male Sprague-Dawley rats.

## Methods

### Sample preparation

Brown rice (*Oryza sativa* MR220 & MR219), varieties that commonly available in the market, was obtained from Padiberas Nasional Berhad (Bernas), Malaysia. The rice was germinated according to the pre-optimized conditions established in Laboratory of Molecular Biomedicine, Institute of Bioscience, Universiti Putra Malaysia (UPM), Selangor, Malaysia as described previously [24]. The GBR was ground to powder using a stainless steel blender (Waring Commercial, Torrington, CT, USA) before used to make rat pellets. Based on our previous study, GBR powder (per 100 g sample) was contained of moisture, 14.04 g; fat, 2.11 g; protein, 11.03 g; carbohydrate, 54.30 g; dietary fiber, 9.18 g, and its energy content was 390.95 ± 11.311 kcal [25].

### Animals

Forty-six male Sprague-Dawley rats (166.02 ± 19.63 g) were individually housed in polycarbonate cages (15 cm x 25 cm) with stainless steel covers in the animal house of Faculty of Medicine and Health Sciences, UPM with controlled conditions (24 ± 2 °C, 85 % relative humidity and a controlled 12 h light-dark cycle) throughout the experiment. Male rats were selected to eliminate variations in food intake due to ovarian hormones [26], in addition to their faster-growing degree than females, which enable easier detection of changes in body weight [27]. All rats were acclimatized for a week with the commercial food pellets (Gold Coin, Port Klang, Malaysia). The food pellet consisted of 13 % moisture, 8 % ash, 50 % carbohydrate, 21 % protein, 3 % fat and 5 % fiber. Diets and distilled water were provided *ad libitum* to rats all through the experiment. Experimental procedures were obtained ethical clearance with reference No. UPM/IACUC/AUP-R034/2014 and were conducted in accordance with the guidelines established by the Institutional Animal Care and Use Committee of UPM.

### Induction of obesity

Following acclimatization, the rats were randomly divided into normal diet control (NC) group (*n* = 11) and HFD group (*n* = 35). The NC group was continually on commercial food pellets while the latter group was induced obesity by feeding HFD for 8 weeks. The HFD was formulated based on Levin et al. [28] with modification. It was prepared from a mixture of 50 % commercial food pellet, 24 % ghee, 20 % full-cream milk powder and 6 % corn starch. HFD was prepared weekly, to avoid spoilage, by mixing all ingredients thoroughly, spread in trays, cut into smaller pieces and placed in an oven at 65 °C for 24 h. It was stored at 4 °C to avoid lipid oxidation. After the induction period, the mean body weights of the HFD group rats were compared with the NC group. Rats with more than 10 % body weights than the maximum body weights of normal diet rats were considered as obese [29]. The obesity was also confirmed by using Lee index. It was calculated by the cube root of body weight (g)/nose-anal length (cm), for which a value equal to or lower than 0.30 was classified as normal. For value higher than 0.30, the rat was classified as obese [30]. Three rats from NC group and HFD groups, respectively, were sacrificed under anesthesia to obtain data on their weight of adipose tissue. The adipose tissue of HFD group rats should weigh 30–45 % more than those of NC group rats [29].

### Treatments of GBR

After confirming that the HFD rats were obese (8 weeks of obesity induction), the rats were further subdivided into HFD positive control (PC) group (*n* = 8), HFD-induced obese rat administrated with 25 % GBR (25 T) group (*n* = 8), HFD-induced obese rat administrated with 50 % GBR (50 T) group (*n* = 8), and HFD-induced obese rat administrated with 100 % GBR (100 T) group (*n* = 8). Each of the group was provided with their respective diets for another 8 weeks. The NC group (*n* = 8) was continually on commercial food pellets, the PC group was on the formulated HFD, the 25 T group was received formulated HFD in which 25 % of the commercial food pellets was substituted with GBR, the 50 T group was received formulated HFD in which 50 % of the commercial food pellets was substituted with GBR, and the 100 T group was received formulated HFD in which all of the commercial food pellets was substituted with GBR. Treatment diets were prepared in a similar way as formulated HFD. The energy content of each diet was determined by bomb calorimetry (IKA C5003, IKA Werke, Germany) according to the manufacturer’s protocol and expressed as kcal/100 g of sample.

### Anthropometric and dietary measurements

The body weight of each rat was measured at weekly intervals and food intake was recorded twice in a week using electrical balance. The BMI was calculated as body weight (g) divided by the square of the anal-nasal length (cm). The feed efficiency was calculated as weight gain per day (g) divided by either food intake per day (g) or energy intake per day (kcal).

### Blood and organ tissue collection

Blood samples were collected after an overnight fast under ketamine and xylazine anesthesia during the end of the induction and end of the treatment. The collected blood was centrifuged for 10 min at 3500 rpm at room temperature and the supernatants were collected and kept at −80 °C. At the end of the treatment, rats were exsanguinated through cardiac puncture. The liver was surgically removed, rinsed with physiological saline solution and wet weights were measured with an analytical balance after blotting. The white adipose tissues (abdominal, epididymal and perirenal) were dissected and wet weights were recorded.

### Biochemical analysis

Plasma of total cholesterol, triglyceride and high-density lipoprotein (HDL) cholesterol were measured using enzymatic colorimetric method (Cobas 8000, Roche-Diagnostics, Basel, Switzerland). Fasting blood glucose level was analyzed by UV-Test with hexokinase reference method (Cobas 8000, Roche-Diagnostics, Basel, Switzerland). Plasma leptin concentration was quantified using the enzyme-linked immunosorbent assay (ELISA) kits according to as described in the manufacturer’s instruction (Catalog No. CSB-E07433r, CUSABIO BIOTECH CO., LTD, China).

### Fecal fat content analysis

Feces were collected from rats on 7 consecutive days on the last week of obesity induction and on the last week end of the treatment. They were freeze dried and ground by mortar and pestle before being analyzed using a soxhlet extraction apparatus (Soxtec Avanti 2050, Foss Tecator AB, Hoganas, Sweden) with petroleum ether. The fecal fat content is expressed as the percentage of fat.

### Histopathological studies of liver and white adipose tissue

The liver and white adipose tissues were fixed in a buffer solution of 10 % formalin. The fixed tissues were processed routinely for paraffin embedding, after which 4–5 μm sections were prepared and stained with hematoxylin and eosin (H&E). The stained areas were then viewed using an optical microscope.

### Statistical analysis

Data are expressed as means ± standard deviations (SD). Analyzes were performed by one-way analysis of variance (ANOVA), followed by Duncan’s multiple-range test, using IBM SPSS software version 21.0. Paired sample *T*-test (two tails) was performed to compare the results obtained before treatment and after treatment within same groups whereas Independent sample *T*-test (two tails) was performed to compare the results obtained between two groups. Values were considered statistically significant when *p* < 0.05.

## Results

### Energy distribution of each diet

Table 1 shows the energy distribution in the percentage of different diets fed to the rats. HFD indicated the highest energy content (560.13 ± 4.17 kcal/100 g) and was used to induce obesity among HFD group for 8 weeks. Energy contributed from fat sources in HFD diet was 52.0 kcal% and it was 43 % increase fat composition compared with normal diet (9.0 kcal%). The energy content of the normal diet was only 389.74 ± 1.11 kcal/100 g in which carbohydrate was contributed the highest energy with 64.0 kcal%. For treatment groups, energy content was 532.58 ± 9.91 kcal/100 g for 25 T (HFD + 25 % GBR), 528.63 ± 4.51 kcal/100 g for 50 T (HFD + 50 % GBR) and 544.30 ± 4.03 kcal/100 g for 100 T (HFD + 100 % GBR). Energy contributed by fat was 52.0 kcal% in all treatment diets.

**Table 1:** Energy distribution in percentage of each diet in the experiment

Type of diet^1^	Normal diet^2^	HFD	HFD + 25 % GBR	HFD + 50 % GBR	HFD + 100 % GBR
Protein (kcal%)	27.0	13.0	13.0	12.0	10.0
Carbohydrate (kcal%)	64.0	35.0	35.0	36.0	38.0
Fat (kcal%)	9.0	52.0	52.0	52.0	52.0
Total energy (kcal/100 g)^2^	389.74 ± 1.11^a^	560.13 ± 4.17^b^	532.58 ± 9.91^c^	528.63 ± 4.51^c^	544.30 ± 4.03^d^

Values with different superscripts in a row indicate significant difference (*p* < 0.05) by Duncan’s multiple range tests

*HFD* high fat diet, *GBR* germinated brown rice

^1^Values are calculated from total energy while total energy was determined by bomb calorimetry (IKA C5003, IKA Werke, Germany)

^2^Commercial food pellets from Gold Coin, Port Klang, Malaysia

### Anthropometry, dietary status, and weight of organs and white adipose tissue measurement during obesity induction period

The anthropometry, dietary status, and weight of organs and white adipose tissue during obesity induction period are shown in Table 2. The body weight gains of the HFD group were significantly higher (*p* < 0.05) than the NC group although their body weights were similar at the onset of the experiment. Similarly, significantly higher (*p* < 0.05) Lee index and BMI were observed in the HFD group compared with the NC group. No significant difference in food intake was observed throughout the obesity induction period in both groups. However, the energy intake and feed efficiency indicated significantly higher value (*p* < 0.05) in the HFD group compared with the NC group.

**Table 2:** Anthropometry, dietary status, and weight of organs and white adipose tissues during obesity induction period by high fat diet in rats

**Groups**	**NC** ^**1**^	**HFD** ^**2**^
Body weight (g)		
Initial	221.36 ± 24.33^a^	218.86 ± 21.10^a^
Final	335.09 ± 19.28^a^	420.70 ± 41.71^b^
Body weight gain (g)	113.73 ± 15.88^a^	202.61 ± 34.50^b^
Lee index	0.29 ± 0.00^a^	0.31 ± 0.00^b^
Body mass index	0.60 ± 0.02^a^	0.70 ± 0.04^b^
Food intake (g/day/rat)	15.80 ± 4.48^a^	16.44 ± 1.10^a^
Energy intake (kcal/day/rat)	61.63 ± 17.46^a^	89.03 ± 7.27^b^
Feed efficiency (g/g)	0.13 ± 0.02^a^	0.22 ± 0.04^b^
Feed efficiency (g/kcal)	0.03 ± 0.00^a^	0.04 ± 0.01^b^
**Groups**	**NC** ^**3**^	**HFD** ^**3**^
Organ weight (g/100 g BW)		
Liver	2.26 ± 0.05^a^	2.75 ± 0.21^a^
Kidney	0.32 ± 0.01^a^	0.33 ± 0.00^a^
White adipose tissue weight (g/100 g BW)	0.66 ± 0.09^a^	4.86 ± 0.25^b^
Abdominal	0.58 ± 0.02^a^	2.69 ± 0.29^b^
Epididymal	0.53 ± 0.13^a^	1.69 ± 0.17^b^
Perirenal	0.19 ± 0.05^a^	0.48 ± 0.07^b^

Values with different superscripts in a row indicate significant difference (*p* < 0.05) by Independent-Samples *T* test

*NC* normal diet, *HFD* high-fat diet

^1^ Values are expressed as means ± SD (*n* = 11)

^2^ Values are expressed as means ± SD (*n* = 35)

^3^ Values are expressed as means ± SD (*n* = 3)

No significant difference was observed in liver and kidney weights normalized to 100 g of body weight between the HFD group and the NC group. However, with regard to white adipose tissue, the weights of abdominal, epididymal and perirenal in rats fed on HFD were significantly higher (*p* < 0.05) compared with the NC group rats. The total white adipose tissue weights of rats fed on HFD was increased 86.42 % compared with the NC group rats. The adipose tissue weight gains of abdominal, epididymal and perirenal were increased 78.44, 43.12, and 10.78 % compared with the NC group, respectively.

### Anthropometry, dietary status, and weight of organs and white adipose tissue measurement during treatment period

**Fig. 1:**
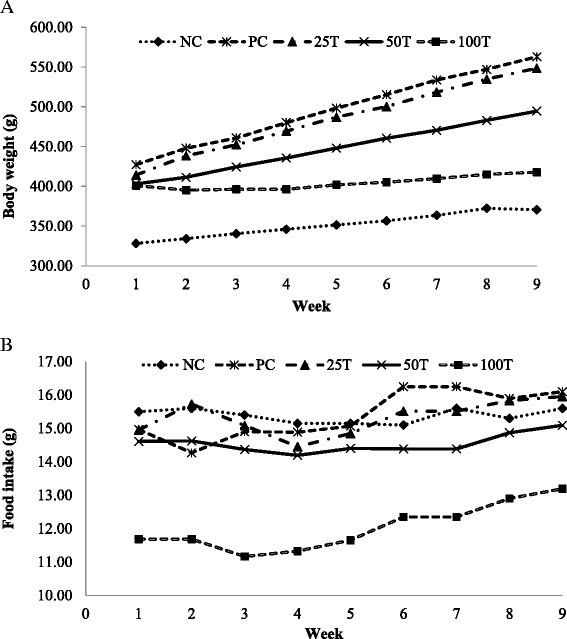
Effects of GBR on body weight and food intake of rats during the 8 weeks treatment period. **a** Changes of body weight. **b** Changes of food intake. Values are expressed as means ± SD (*n* = 8). NC, normal diet; PC, high-fat diet; 25 T, high-fat diet + 25 % GBR; 50 T, high-fat diet + 50 % GBR; 100 T, high-fat diet + 100 % GBR

Figure 1 (a) shows the changes of body weights of rats during the 8 weeks treatment period. The body weights of the rats increased gradually during the treatment period in all groups except in the 100 T group where the body weights in this group were relatively consistent. GBR administration for 8 weeks significantly decreased (*p* < 0.05) body weight gains in the 100 T group (16.88 ± 10.12 g) and 50 T group (91.50 ± 23.24 g) as compared with the PC group (135.63 ± 34.04 g) as shown in Table 3. The Lee index and BMI in these two groups were also significantly lower (*p* < 0.05) than the PC group. The mean body weight gains in the 100 T group were notably lower compared with the NC group although they were fed on HFD. The changes in food intakes during the treatment period are shown in Fig. 1 (b). Similarly, the food intakes were significantly lower (*p* < 0.05) in the 50 T group and the 100 T group compared with other groups. There was no significant difference in food intakes between the PC group (15.29 ± 1.11 g) and the NC group (15.38 ± 1.02 g). At the same time, the administration of GBR significantly reduced (*p* < 0.05) energy intake in the 25 T, 50 T, and the 100 T group as compared with the PC group in a dose-dependent manner although all of these groups were fed on HFD. The feed efficiency of the 50 T group and the 100 T group were significantly lower (*p* < 0.05) than the PC group but the feed efficiency of the 25 T group was comparable with the PC group.

**Table 3:** Anthropometry, dietary status, and weight of organs and white adipose tissues during GBR treatment period in diet-induced obese rats

Groups	NC	PC	25 T	50 T	100 T
Body weight (g)					
Initial	328.25 ± 23.30^a^	427.25 ± 57.91^b^	414.25 ± 27.91^b^	403.00 ± 33.56^b^	400.88 ± 29.68^b^
Final	370.63 ± 29.12^a^	562.88 ± 85.62^b^	548.50 ± 43.27^bc^	494.50 ± 59.58^c^	417.75 ± 45.47^a^
Body weight gain (g)	42.38 ± 14.73^a^	135.63 ± 34.04^b^	134.25 ± 20.49^b^	91.50 ± 23.24^c^	16.88 ± 10.12^a^
Lee index	0.29 ± 0.00^a^	0.32 ± 0.01^b^	0.31 ± 0.00^b^	0.31 ± 0.01^c^	0.30 ± 0.01^a^
Body mass index	0.62 ± 0.03^a^	0.82 ± 0.06^b^	0.81 ± 0.04^b^	0.74 ± 0.06^c^	0.67 ± 0.06^d^
Food intake (g/day/rat)	15.38 ± 1.02^a^	15.29 ± 1.11^a^	15.30 ± 0.73^a^	14.57 ± 0.61^b^	12.00 ± 0.86^c^
Energy intake (kcal/day/rat)	59.96 ± 4.00^a^	85.64 ± 6.21^b^	81.53 ± 3.89^c^	77.07 ± 3.24^d^	65.26 ± 4.67^e^
Feed efficiency (g/g)	0.06 ± 0.02^a^	0.16 ± 0.04^b^	0.16 ± 0.02^b^	0.11 ± 0.03^c^	0.04 ± 0.01^a^
Feed efficiency (g/kcal)	0.01 ± 0.00^a^	0.03 ± 0.01^b^	0.03 ± 0.00^b^	0.02 ± 0.01^ab^	0.01 ± 0.00^a^
Organ weight (g/100 g BW)					
Liver	2.43 ± 0.21^a^	2.89 ± 0.20^b^	2.84 ± 0.23^b^	2.82 ± 0.20^b^	2.70 ± 0.25^b^
Kidney	0.46 ± 0.04^ab^	0.47 ± 0.02^b^	0.44 ± 0.03^ab^	0.44 ± 0.04^ab^	0.43 ± 0.03^a^
White adipose tissue weight (g/100 g BW)	1.45 ± 0.91^a^	5.75 ± 1.18^b^	5.59 ± 0.47^bc^	4.97 ± 1.28^bc^	1.31^c^
Abdominal	0.67 ± 0.55^a^	3.51 ± 0.65^b^	3.44 ± 0.40^b^	3.11 ± 0.78^b^	0.99^b^
Epididymal	0.57 ± 0.19^a^	1.71 ± 0.51^b^	1.70 ± 0.31^b^	1.42 ± 0.53^b^	1.28 ± 0.50^b^
Perirenal	0.21 ± 0.07^a^	0.53 ± 0.13^b^	0.45 ± 0.10^b^	0.43 ± 0.17^b^	0.40 ± 0.13^ab^

Values are expressed as means ± SD (*n* = 8)

Values with different superscripts in a row indicate significant difference (*p* < 0.05) by Duncan’s multiple range tests

*NC* normal diet, *PC* high-fat diet, *25 T* high-fat diet + 25 % GBR, *50 T* high-fat diet + 50 % GBR, *100 T* high-fat diet + 100 % GBR

The liver weights of the HFD groups (PC, 25, 50, and 100 T) were significantly higher compared with the NC group. The rats administrated with GBR were showed lower liver weights than the PC group although no significant difference was observed among them. The kidney weights were comparable between the HFD groups and the NC group. Rats fed on HFD showed significant increased (*p* < 0.05) in weight of all three types of white adipose tissues compared with the NC group. Only rats fed with the highest dose of GBR (100 T) was showed a significantly lower amount of total white adipose tissue (4.55 ± 1.31 g/100 g BW) compared with the PC group (5.75 ± 1.18 g/100 g BW). However, no significant difference was observed in individual weight of abdominal, epididymal and perirenal adipose tissue between the 100 T group and the PC group.

### Changes of biochemical analysis

Table 4 shows the changes of plasma lipid profiles, glucose levels and leptin levels between induction and treatment period in rats. Rats fed on HFD for 8 weeks demonstrated significant elevation (*p* < 0.05) of total cholesterol levels and triglyceride levels compared with the NC group. However, the feeding of GBR to obese rats (25, 50, and 100 T) resulted in significant reduction (*p* < 0.05) of the total cholesterol levels as compared with the PC group and with their respectively total cholesterol levels before treatment. The triglyceride levels were significantly lower (*p* < 0.05) in the 50 T group (90.67 ± 11.99 mg/dL) and 100 T group (87.91 ± 11.78 mg/dL) compared with the PC group (143.59 ± 20.60 mg/dL). However, the reduction of triglyceride levels was not significantly difference compared with their respective triglyceride levels before treatment. Although the administration of GBR in obese rats did not increase HDL-cholesterol levels, it was observed that the administration of GBR reduced the declination of plasma HDL-cholesterol levels with only 15.02 % reduction in the 50 T group and 12.66 % reduction in the 100 T group compared with 33.00 % reduction in the PC group. Overall, the administration of GBR improved the plasma lipid profiles in treated obese rats, which was also shown by an increment of HDL-cholesterol/total cholesterol ratio (HTR) compared with untreated obese rats. The HTR was found to be increased in the 50 T group (5.00 %) and the 100 T group (8.65 %) after GBR treatment while the HTR were decreased in the NC group ( −3.62 %), 25 T group ( −3.90 %) and PC group ( −50.97 %) after treatment. In addition, the atherogenic index (AI) was found to be significantly decreased (*p* < 0.05) in all GBR administrated groups compared with the PC group. A significant reduction (*p* < 0.05) of AI was observed in the 50 T group and 100 group compared with their respective AI before treatment. On the other hand, there was no significant difference in glucose levels between induction and treatment in obese rats fed with GBR (25, 50, and 100 T). However, the glucose levels of the NC group and PC group were found to increase significantly (*p* < 0.05) compared with their respectively glucose levels before treatment. Thus, this indicated that GBR might help to mitigate the effect of HFD on glucose level. Besides, the feeding of HFD for 8 weeks was significantly increased (*p* < 0.05) the leptin levels in the HFD group compared with the NC group. The leptin levels were found to be decreased but not significantly different in the treatment groups (25, 50, and 100 T) compared with their respectively leptin levels before treatment. Oppositely, the leptin levels were found to be increased in the NC group and the PC group after treatment.

**Table 4:** Changes of plasma lipid profile, glucose level and leptin level between induction and treatment period in rats

Group		Total cholesterol (mg/dL)	Triglyceride (mg/dL)	HDL-cholesterol (mg/dL)	HTR (%)^1^	AI^2^	Glucose level (mg/dL)	Leptin level (ng/mL)
NC	Induction	53.61 ± 9.56^Aa^	46.94 ± 9.19^Aa^	40.02 ± 4.63^Aa^	75.48 ± 6.38^Aa^	0.33 ± 0.12^Aa^	121.62 ± 7.64^Aa^	3.59 ± 0.42^Aa^
	Treatment	57.57 ± 10.64^Aa^	59.56 ± 7.33^Ba^	40.70 ± 6.01^Aa^	71.77 ± 9.91^Aa^	0.35 ± 0.11^Aa^	130.18 ± 5.76^Ba^	3.65 ± 0.43^Aa^
PC	Induction	66.80 ± 7.48^Ab^	99.53 ± 12.67^Ab^	46.55 ± 8.75^Aab^	69.28 ± 6.56^Aab^	0.45 ± 0.14^Aa^	125.90 ± 12.61^Aa^	4.65 ± 0.62^Ab^
	Treatment	76.37 ± 7.95^Bb^	143.59 ± 20.60^Bb^	35.00 ± 7.10^Ba^	45.89 ± 8.79^Bb^	1.17 ± 0.39^Bb^	138.29 ± 10.93^Bb^	4.97 ± 0.75^Ab^
25 T	Induction	65.40 ± 9.79^Ab^	96.10 ± 14.90^Ab^	48.77 ± 9.75^Ab^	74.34 ± 7.17^Aab^	0.39 ± 0.16^Aa^	115.77 ± 14.41^Aa^	4.78 ± 0.55^Ab^
	Treatment	49.40 ± 5.47^Ba^	127.43 ± 7.20^Bc^	34.85 ± 3.06^Ba^	71.44 ± 11.42^Aa^	0.45 ± 0.15^Aa^	114.64 ± 4.30^Ac^	4.57 ± 0.35^Ab^
50 T	Induction	72.55 ± 11.55^Ab^	100.42 ± 9.68^Ab^	47.03 ± 5.41^Aab^	65.89 ± 10.79^Ab^	0.45 ± 0.18^Aa^	117.79 ± 8.88^Aa^	4.63 ± 0.51^Ab^
	Treatment	59.60 ± 12.70^Ba^	90.67 ± 11.99^Ad^	40.89 ± 9.74^Aa^	69.36 ± 14.19^Aa^	0.35 ± 0.18^Aa^	116.67 ± 2.68^Ac^	4.44 ± 0.60^Ab^
100 T	Induction	72.84 ± 6.51^Ab^	99.75 ± 11.08^Ab^	46.89 ± 5.12^Aab^	65.09 ± 10.72^Ab^	0.49 ± 0.19^Aa^	113.29 ± 11.49^Aa^	4.68 ± 1.02^Ab^
	Treatment	57.86 ± 9.19^Ba^	87.91 ± 11.78^Ad^	41.62 ± 10.03^Aa^	71.25 ± 9.59^Aa^	0.32 ± 0.07^Aa^	112.61 ± 4.41^Ac^	4.33 ± 0.73^Ab^

*NC* normal diet, *PC* high-fat diet, *25 T* high-fat diet + 25 % GBR, *50 T* high-fat diet + 50 % GBR, *100 T* high-fat diet + 100 % GBR

^1^HTR = (HDL-cholesterol/total cholesterol) × 100

^2^ *AI* atherogenic index = (total cholesterol - HDL-cholesterol)/HDL-cholesterol

Block letters indicate significant difference (*p* < 0.05) within the same group at different time intervals by Paired sample *T*-test, while lower case letters indicate significant difference (*p* < 0.05) between groups regarding the corresponding time interval by Duncan’s multiple range tests

### Fecal fat content analysis

**Fig. 2:**
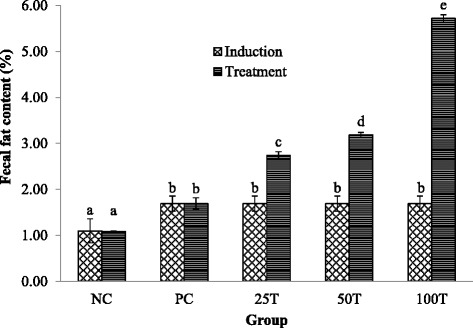
Fecal fat content for obesity induction period and treatment period. Values are expressed as means ± SD (*n* = 3). Values with different superscripts indicate significant difference (*p* < 0.05) by Duncan’s multiple range tests. NC, normal diet; PC, high-fat diet; 25 T, high-fat diet + 25 % GBR; 50 T, high-fat diet + 50 % GBR; 100 T, high-fat diet + 100 % GBR

Figure 2 shows the fecal fat content of rats during obesity induction period and treatment period. No significant difference in the fecal fat content of rats was observed between induction and treatment period for the NC group and the PC group. However, the fecal fat content was found to be increased significantly (*p* < 0.05) in rats administrated with GBR in a dose-dependent manner compared with the PC group and their respective fecal fat content during induction. The fecal fat content was found to be the highest in the 100 T group (5.72 **±** 0.08 %), followed by 50 T group (3.18 **±** 0.05 %) and 25 T group (2.74 **±** 0.08 %).

### Histopathological studies of liver and white adipose tissues

**Fig. 3:**
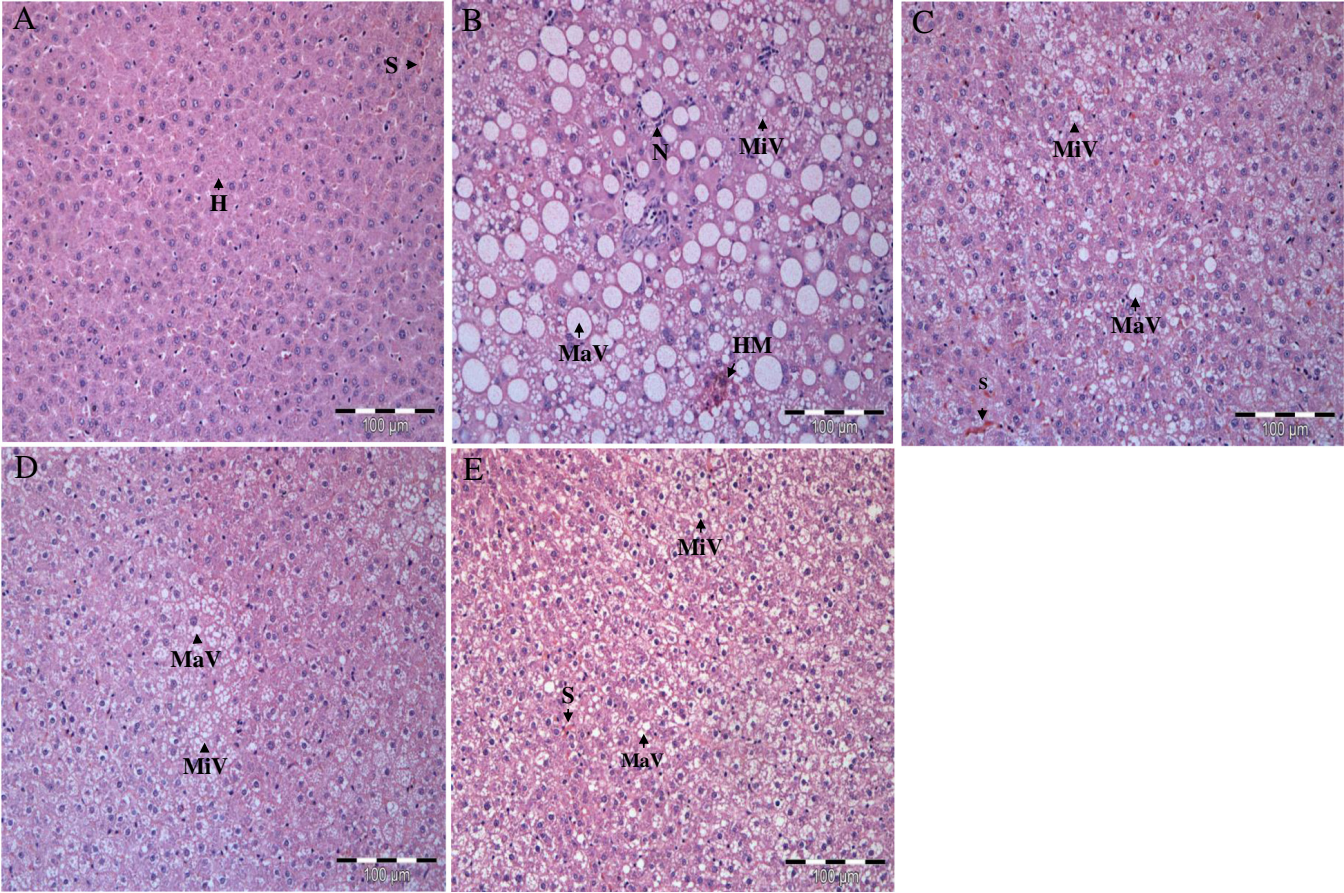
Photomicrographs of liver (Haematoxylin & Eosin, Original magnification x 100) of treated and untreated rats. **a** Normal liver in NC group. **b** Diffuse micro- and macrovesicular steatosis, swollen hepatocytes, and hepatocyte necrosis was evidenced in PC group. **c–e** The treatments of GBR reduced micro- and macrovesicular steatosis and other pathological features was witnessed in 25, 50 and 100 T group. H, Hepatocytes; MaV, Macrovesicular; MiV, Microvesicular; N, Neutrophils; S, Sinusoids

As shown in Fig. 3(a), the liver of NC group showed normal lobular architecture with normal hepatocytes arranged in one cell plate separated by sinusoids. GBR administration reduced micro- and macrovesicular steatosis as compared with untreated obese rats. The liver of the PC group exhibited severe and diffuse micro- and macrovesicular steatosis (Fig. 3(b)), whereas mild and focal micro- and macrovesicular steatosis were observed in the obese rats administrated with GBR (Fig. 3(c–e)). Necrosis was also evidenced in the PC group but none in the GBR administrated obese rats. No hepatitis, hemorrhage, and perivenular fibrosis were observed in all rats. Overall, histopathological analysis and their qualitative scoring are summarized in Table 5.

**Table 5:** Histopathological scoring of liver in normal weight rats and HFD-induced obese rats with and without treatment of GBR for 8 weeks

Parameter^a^	NC	PC	25 T	50 T	100 T
Hepatic steatosis					
Microvesicular	-	+++	++	++	++
Macrovesicular	-	+++	++	++	++
Hepatitis	-	-	-	-	-
Haemorrhage	-	-	-	-	-
Hepatocyte necrosis	-	+	-	-	-
Fat cysts	-	-	-	-	-
Perivenular fibrosis	-	-	-	-	-

*NC* normal diet, *PC* high-fat diet, *25 T* high-fat diet + 25 % GBR, *50 T* high-fat diet + 50 % GBR, *100 T* high-fat diet + 100 % GBR

^a^The severity was evaluated based on the following scoring scheme: *- normal, + mild effect, ++ moderate effect, +++ severe effect*

**Fig. 4:**
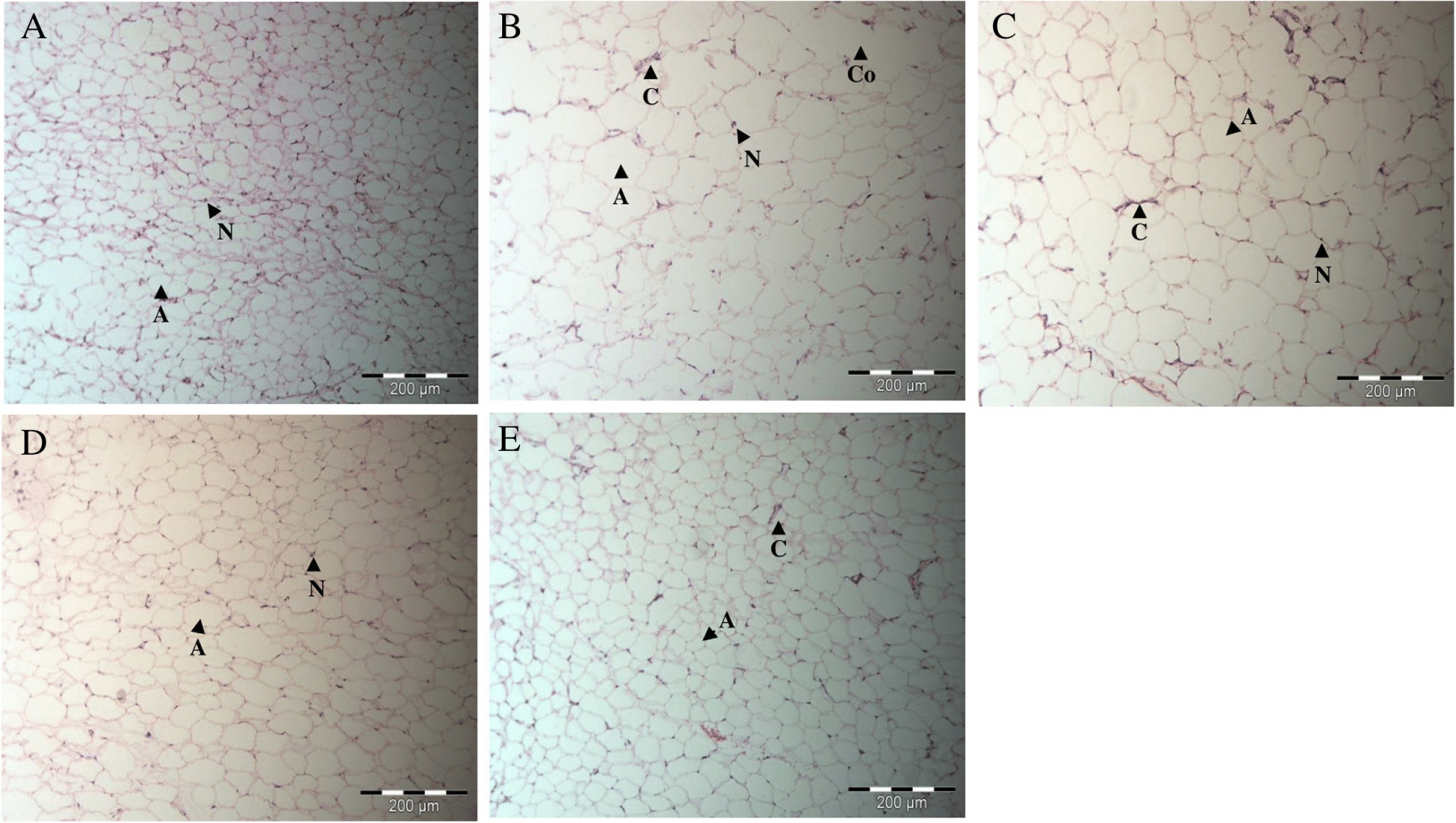
Photomicrographs of white adipose tissue (Haematoxylin & Eosin, Original magnification x 200) of treated and untreated rats. **a** Smaller size of adipocytes was clearly observed in NC group. **b** Larger size of adipocytes was seen in PC group. The enlarged adipocytes might due to coalescence of adjacent adipocytes cells. **c–e** The size of adipocytes in the obese rats treated with GBR was clearly smaller in 25, 50 and 100 T group. A, Adipocytes; C, Blood capillaries; Co, Coalescence; N, Nucleus

White adipose tissues of the various groups of rats are shown in Fig. 4(a–e). Routine processing methods for microscopy generally extract lipid from tissues leaving the lipid droplets within cells appear as unstained vacuoles. The white adipose tissue exhibited larger adipocytes in the PC group (Fig. 4(b)), whereas the NC group showed normal adipocytes distribution with regular sizes of cells (Fig. 4 (a)). The fat stored in adipocytes in obese rats especially the not administrated rats were accumulated as lipid droplets which fuse to form a single large droplet which distends and occupies most of the cytoplasm. However, the adipocytes of obese rats treated with GBR indicated smaller sizes and similar histology to the NC group especially in the 100 T group rats (Fig. 4(e)). No acute and chronic inflammatory infiltrates or necrosis was seen in all rats.

## Discussion

This present study demonstrated that the HFD intake confirms induction of obesity in rats. Numerous comprehensive review of past studies have summarized high-fat food significantly upsurge accumulation of adipose tissue, due to their high energy density, leading to increasing of body weight [31–33]. In this study, it was observed that GBR treatment exerted beneficial effects against obesity. Body weight gains and food intakes were found to be significantly reduced in GBR administrated obese rats (50 and 100 T), resulted in a reduction of the Lee index and BMI. Similar results have been reported by Ho et al. [9], in which mice supplemented with 0.15 % GBR methanol extract showed a significant reduction in their weight gain. However, they found no significant reduction in food intake compared with untreated mice for 7 weeks. Oh et al. [10] also found a reduction in weight gain and food intake in mice fed with 10 μl/g body weight of GBR water extract for 8 weeks. The differences between this present study and these two previous studies are the approaches used, whereby the GBR was given as a whole food after rats were induced with obesity and further continued on HFD. Besides, GBR was showed containing a significantly higher amount of dietary fiber than white rice and brown rice [18]. The bulking and viscosity properties of dietary fiber are able to suppress food intake by promoting satiation and satiety. Increasing efforts and/or time of mastication in fiber-rich foods lead to increase satiety through a reduction in the rate of ingestion [34]. The promotion of satiety by GBR, as a whole grain food, is elucidating the reduction of food intake in GBR administrated obese rats.

Apart from that, the treatment of GBR also significantly lowered the plasma total cholesterol and triglyceride levels. The HDL-cholesterol level showed a reduction after administration of GBR. However, the percentage of reduction in the HDL-cholesterol level was lesser in GBR treated obese rats compared with untreated obese rats. One of the possible reason for the reduction in HDL-cholesterol level among GBR treated obese rats might be due to the continuation feeding of HFD during the treatment period as several studies showed that HFD resulted in dyslipidemic changes by increasing levels of triglyceride, total cholesterol, LDL-cholesterol and reducing level of HDL cholesterol [35, 36]. In addition, there were few studies reported similar results that GBR decreased the concentration of blood total cholesterol and triglycerides compared with untreated HFD group [9, 10, 37]. Ho et al. [9] postulated that the GABA, vitamin E, and γ-oryzanol in GBR were the components that exerted hypocholesterolemia in mice fed on HFD. Thus, this may explain why the administration of GBR was found to be increased the HTR but decreased the AI in GBR administrated obese rats. Besides, the reduction of the total cholesterol and triglyceride is also believed to be associated with dietary fibers through the increment of steroid and bile acid excretion into feces. Stronger bile acids binding ability of rice bran compared with oat bran, wheat bran, and corn bran has been reported [38]. The previous study hypothesized the cholesterol-lowering mechanism of pre-GBR might be due, at least in part, to the increment of steroid and bile acid excretion into feces with an enhancement in cholesterol 7α-hydroxylase activity in the liver microsomal fraction [39].

In this study, administrated of GBR reduced levels of leptin in the GBR administrated obese rats. It is more likely that reduction of plasma leptin was due to the decreased of the white adipose tissue mass. This is because blood leptin levels are closely related to fat deposition [40]. Previous studies have shown similar results that decreased of fat mass resulted in a reduction in leptin concentrations [41, 42]. The results of this study indicated that the administration of GBR resulted in decreased of white adipose tissues. In addition, obesity is closely correlated with insulin resistance and hyperglycemia [43]. Surprisingly, the glucose levels of obese rats were not significantly different than normal weight rats in this study. Other previous studies had also reported similar findings in which they found no significant difference in blood glucose between normal weight rats and obese rats [13, 44]. However, they found insulin resistance in obese rats. This is consistent with the notion that obesity is often associated with insulin resistance [43]. On the other hand, Imam et al. [17] reported a hypoglycemic effect of GBR on streptozotocin-induced diabetes rats. Thus, further study is warranted to better elucidate the effect of GBR on insulin resistance and hyperglycemia under both physiological and pathophysiological conditions.

In this present study, the GBR treatment enhanced the fat excretion into feces in GBR administrated obese rats. It is possible that anti-obesity effects of GBR, which might account, at least partially resulted from suppressing dietary fat digestion via inhibition of pancreatic lipase activity with subsequent excretion of the undigested fat, which was consistent with the findings of the in vitro experiment [45]. Furthermore, this present study showed a significant reduction in total white adipose tissue mass (abdominal, epididymal and perirenal) and shrinkage in adipocytes in GBR fed obese rats. It is plausible that GBR exerts its anti-obesity effects by decreasing fat accumulation through inhibiting adipocyte differentiation and stimulating lipolysis on adipocytes as reported in the in vitro study [45]. This is further supported by the down-regulated expression of adipogenic transcription factors and adipogenic genes in 3T3-L1 adipocytes studied in GBR extract previously [9]. Moreover, micro- and macrovesicular steatosis was evidently attenuated in GBR treated obese rats. The consumption of HFD contributed to obesity, which is increased the risk of nonalcoholic fatty liver disease in obese individuals [46]. Steatosis, the hallmark of a feature of the nonalcoholic fatty liver disease, progresses to steatohepatitis, eventually to cirrhosis [47]. The treatment of GBR results in a reduction of steatosis, suggesting the potential role of GBR in restoring the liver damage caused by HFD.

## Conclusions

In conclusion, this study provides evidence that GBR ameliorates obesity by suppressing body weight gain and food intake while improving lipid profiles and reducing leptin level and white adipose tissue mass in obese rats fed on HFD. The beneficial effects of GBR on obesity seem to be partly attributed to the synergistic effects of its high dietary fibers, vitamins, minerals and other bioactive compounds such as GABA, γ-oryzanol, phytosterols, polyphenols, tocotrienols and α-tocopherol. However, further studies are required to verify the effectiveness of GBR in fighting obesity in human study and to recommend the dosage to use GBR in the daily diet.

## Abbreviations

25 T: HFD-induced obese rat administrated with 25 % GBR
50 T: HFD-induced obese rat administrated with 50 % GBR
100 T: HFD-induced obese rat administrated with 100 % GBR
ANOVA: one-way analysis of variance
BMI: body mass index
ELISA: enzyme-linked immunosorbent assay
GABA: γ-aminobutyric acid
GBR: germinated brown rice
H&E: hematoxylin and eosin
HDL: high density lipoprotein
HFD: high-fat diet
NC: normal diet control
PC: HFD positive control
SD: standard deviations
UPM: Universiti Putra Malaysia
